# Supplementary material for: Antimicrobial Properties of Compounds Isolated from Syzygium malaccense (L.) Merr. and L.M. Perry and Medicinal Plants Used in French Polynesia
Source: Life (Basel). 2022 May 14;12(5):733. doi: 10.3390/life12050733 (PMC9147835; doi:10.3390/life12050733)
Supplement: Supplementary file 1 [file life-12-00733-s001.zip › life-1721674-supplementary.pdf]

Supplementary data

Table S1. Medicinal plants cited in the survey but not collected, with their medicinal uses.

Parts used: leaf (l.); fruit (f.); flower (fl.); bud (b.); bark (bk.); whole plant (w.p.); rhizome (rh.); root (r.); aerial part (a.p.); aerial root (a.r.); stem (st.); almond (alm.); latex of fig (l.f.). Route of administration-preparation: Per os (Po); Cataplasm (Cat); Gargle (Garg); Inhalation (Inh). Medicinal uses (abbreviations and specifications): **V. Cutaneous symptoms** (spots, rash, erysipelas, folliculitis), **Pneumo.** (bronchitis; cough; pneumonia); **V. diseases** (general infectious diseases), **STD** (Sexually transmitted disease: malodorous yellow-greenish genital flow, genital itching…), **Uterus** (care post-partum, "impure" menstruation), **Burn** (sunburn sometimes with headache and dehydration), **Purge** (diet between ra'au tahiti and conventional medicinal drug), **"Crumpled lung"** (crumpled lung provoking cough and pain), **Umbilic.** (care of umbilical cord).

Latin name "vernacular name" (Botanical family) (number of citation), **plant collected in bold**	**Health problem category** (part used; mode of administration)
Achyranthes aspera L. var. *aspera* " 'aerofai" (Amaranthaceae) (3)	**Pneumo.** (l. or w.p.; Po), **Tonsillitis** (b.; Garg and Po)
Acrostichum aureum L. "fa'ato" (Pteridaceae) (1)	**Furunculous, Pustule** (Young frond; Cat)
Artocarpus altilis (Parkinson) Fosberg " 'uru pae'a" (Moraceae). (6)	**Pneumo.** (b. or st.; Po), **Umbilic** (b.; Po and Cat), **Uterus** (b. or st.; Po), **Diarrhoea** (b.; Po), **Furunculous, Pustule** (Sap; Cat), **Sinusitis, Rhinitis** (b.; Po), **Tonsillitis** (bk.; Garg and Po) (st. Garg or Garg and Po)
Capsicum frutescens L. "ma'a 'oporo" (Solanaceae) (2)	**Uterus** (f.; Po), **Pneumo.** (f.; Inh)
Cenchrus caliculatus Cav. "piripiri" (Poaceae) (1)	**V. diseases** (a.p.; Po and Cat)
Citrus × *sinensis* (L.) Osbeck " 'anani" (Rutaceae) (1)	**Pneumo.** (l.; Po), **Leucorrhoea** (l.; Po)
Cocos nucifera L. "ha'ari" (Arecaceae) (5)	**Pneumo.** (Heart (uto) or r.; Po), **Burn** (Milk; Bath), **Purge** (Copra dry; Po), **Sinusitis, Rhinitis** (Copra; Po), **V. diseases** (Copra; Po and Cat)
Cyathula prostrata (L.) Blume "mata'ura" (Amaranthaceae) (2)	**Uterus** (l.; Po), **V. diseases** (w.p.; Po and Cat)
Cyclophyllum barbatum (G.Forst.) N.Hallé & J.Florence "toro'e'a" or "torote'a" (Rubiaceae) (2)	**Conjunctivitis** (l.; In eyes), **Furunculous, Pustule** (b.; Bath)
Cyperus javanicus Houtt. "mo'u" (Cyperaceae) (1)	**Furunculous, Pustule** (Apex of plant; Cat)
Dichrocephala integrifolia (L.f.) Kuntze "ta'ata'ahiara**"** (Asteraceae) (4)	**Pneumo.** (Apex of plant; Po), **Uterus** (Apex; Po), **Heartburn** (Apex; Po), **V. Cutaneous symptoms** (w.p.; Po)

Emilia fosbergii Nicolson<br>“ma'a rapiti” (Asteraceae) (1)	**Wound, Abscess** (l.; Cat)
Gardenia taitensis DC. “tiare tahiti” (Rubiaceae) (6)	**"Crumpled lung"** (b.; Po), **Pneumo.** (b. and/or fl.; Po), **Burn** (fl. and/or b. or l. Bath), **Conjunctivitis** (fl.; In eye), **Cystitis** (fl. and/or b. or l.; Po) **Furunculous, Pustule** (fl. and/or b. or l.; Po or Po and Cat.), **Heartburn** (fl. and/or b.; Po), **Leucorrhoea** (l.; Po), **Sinusitis; Rhinitis** (fl.; Inh), **V. Cutaneous symptoms** (b.; Bath or Cat) (fl. and/or b.; Po and Cat), **Wound, Abscess** (fl. and/or b.; Po and Cat)
Heliotropium arboreum (Blanco) Mabb. “tahinu” (Boraginaceae) (7)	**Acne** (b.; ND), **Pneumo.** (b.; Po), **Tonsillitis** (b.; Garg and Po) (l.; Po)
Hibiscus rosa-sinensis L.<br>“ ‘aute” (Malvaceae) (3)	**Burn** (l.; Bath), **Furunculous, Pustule** (l.; ND), **Wound, Abscess** (b.; Cat)
Leucas decemdentata (Willd.) Sm.<br>“niu” (Lamiaceae) (8)	**Pneumo.** (l. and st.; Po), **Conjunctivitis** (l.; Po), **Furunculous, Pustule** (l. and st.; Po), **Sinusitis, Rhinitis** (l. and st.; Po), **V. Cutaneous symptoms** (w.p.; Po), **V. diseases** (w.p.; Po and Cat)
Mentha spp.<br>“ ‘otime” (Lamiaceae) (1)	**Sinusitis, Rhinitis** (b.; Po)
Momordica charantia L.<br>“fuka” (Cucurbitaceae) (1)	**Mycosis** (Vine; Bath)
Morinda citrifolia L.<br>“nono” or “noni” (Rubiaceae). (5)	**Pneumo.** (f.; Inh), **Burn** (f. without seeds; Cat), **Furunculous, Pustule** (f. with fl.; Cat), **Tonsillitis** (f. with fl.; Po or Po and Cat), **Wound, Abscess** (f. without seeds; Cat)
Ophioglossum reticulatum L. “ti’apito” (Ophioglossaceae) (5)	**Acne** (l.; ND), **Uterus** (l.; Po), **Sinusitis, Rhinitis** (l. or w.p.; Po)
Oxalis corniculata L.<br>“ ‘ahi'a 'ava'ava” (Oxalidaceae) (3)	**Tonsillitis** (l.; Garg and Po or Po) (Vine; Po)
Paspalum orbiculare G.Forst. “ ‘aretu” (Poaceae) (1)	**V. diseases** (w.p.; Po and Cat)
Persicaria glabra (Willd.) M.Gómez “pitorea” (Polygonaceae) (2)	**Pneumo.** (b. or l.; Po)
Premna serratifolia L.<br>“ ‘avaro” (Lamiaceae) (1)	**Constipation** (l.; Po)
Ricinus communis L.<br>“ND” (Euphorbiaceae) (1)	**Wound, Abscess** (alm.; Cat)
Rosa sp. “roti fautau’a” (Rosaceae) (5)	**Sinusitis, Rhinitis** (b. or fl.; Po)
Saccharum officinarum L.<br>“to patu” or “to tore” or “to” (Poaceae) (6)	**Acne** (Internode; ND), **Pneumo.** (Internode; Po), **Umbilic.** (b. or Internode; Po and Cat), **Diarrhoea** (b. or Internode; Po), **Sinusitis; Rhinitis** (Internode; Po)
Schizostachyum glaucifolium (Rupr.) Munro “ 'ofe” (Poaceae) (1)	**V. diseases** (b.; Po and Cat)
Spondias dulcis Parkinson “vi tahiti” (Anacardiaceae) (7)	**Pneumo.** (f. or l.; Po), **Conjunctivitis** (l.; Po or Po and Cat.), **Furunculous, Pustule** (l.; Po), **Oral mycosis** (l.; Po), **Otitis**. (l.; In ear), **V. Cutaneous symptoms** (f.; Po or Po and Cat)

Terminalia catappa L.<br>“ ‘aua” (Combretaceae) (2)	**"Crumpled lung"** (l.; Po), **V. Cutaneous symptoms** (l.; Cat)
Zingiber officinale Roscoe<br>“re’a tinito” (Zingiberaceae) (1)	**STD** (rh.; Po)
Zingiber zerumbet (L.) Roscoe ex Sm.<br>“re’a moruru” (Zingiberaceae) (1)	**Sinusitis, Rhinitis** (rh.; Inh)

Table S2. Determination of antibiotic susceptibility of bacteria (MIC for Minimal Inhibitory Concentration). Gentamycin: S ≤ 4 mg/mL and R > 8 mg/mL; Vancomycin: S ≤ 4 mg/mL and R > 16 mg/mL; Amoxicillin: S ≤ 4 mg/mL et R > 16 mg/mL. S: Sensible, R: Resistant and NA: no activity.

		**CMI (mg/mL)**		
		**Gentamycin**	**Vancomycin**	**Amoxicillin**
Enterococcus faecalis	C159-6	2.0	0,5	64
Enterococcus sp.	8153	2.0	4.0	2.0
Mycobacterium paratuberculosis	5003	0,03	0,5	1.0
Staphylococcus aureus	8146	0,5	1.0	4.0
Staphylococcus aureus	8241	0,5	1.0	16
Staphylococcus aureus	ATCC 6538	0,25	1.0	0,125
Staphylococcus aureus	T28-1	0,5	1.0	2.0
Staphylococcus aureus	T17-4	0,5	1.0	1.0
Staphylococcus epidermidis	T46A1	0,06	2.0	1.0
Staphylococcus epidermidis	T19A1	32	2.0	16
Staphylococcus epidermidis	T21A5	0,06	2.0	16
Staphylococcus warneri	T12A12	0,06	4.0	1.0
Staphylococcus warneri	T26A1	0,06	2.0	0,25
Staphylococcus pettenkoferi	T47.A6	0,06	2.0	0,25
Streptococcus agalactiae	T38.2	NA	NA	NA
Streptococcus agalactiae	T53C9	0,5	0,25	0,03
Streptococcus pyogenes	16138	0,125	0,25	0,03
Streptococcus pyogenes	16135	0,125	0,25	0,03
Corynebacterium striatum	T40A3	0,06	0,5	1.0
Citrobacter freundii	11041	0,25	NA	2.0
Citrobacter freundii	10268	NA	NA	NA
Escherichia coli	ATCC 25922	0,5	NA	16
Escherichia coli	T20A1	0,25	NA	NA
Escherichia coli	8138	0,5	NA	NA

Escherichia coli	8157	0,5	NA	NA
Enterobacter aerogenes	9004	0,5	NA	NA
Klebsiella pneumoniae	10270	0,25	NA	NA
Klebsiella pneumoniae	11016	8	NA	NA
Proteus mirabilis	11060	0,5	NA	2
Proteus mirabilis	T28-3	0,25	NA	1
Pseudomonas aeruginosa	8131	1	NA	NA
Pseudomonas aeruginosa	ATCC 27583	2	NA	NA
Pseudomonas aeruginosa	8129	0,03	NA	NA
Salmonella sp.	11033	0,25	NA	2
